# Analysis of Cytokine Levels in Meibum and Clinical Correlations with Meibomian Gland Dysfunction

**DOI:** 10.1155/2022/4259067

**Published:** 2022-11-23

**Authors:** Wenting Liu, Tong Lin, Lan Gong

**Affiliations:** ^1^Department of Ophthalmology and Vision Science, The Eye, Ear, Nose and Throat Hospital of Fudan University, Shanghai 200031, China; ^2^NHC Key Laboratory of Myopia, Laboratory of Myopia, Chinese Academy of Medical Sciences, Fudan University, Shanghai 200031, China; ^3^Department of Ophthalmology, Huadong Hospital of Fudan University, Shanghai, China

## Abstract

**Objectives:**

This study is aimed at investigating the difference of meibum chemokines in MGD subjects with different degrees of MGD and the correlations of meibum chemokines with ocular surface parameters.

**Methods:**

Twenty MGD subjects (MQ score > 8) and twenty MGD subjects (MQ score ≤ 8) were enrolled to examine ocular surface parameters, including meibomian gland function (MGE, MQ meibograde, and lid margin), tear stability (NIKBUT, FBUT, and LLT), tear secretion (SIT and TMH), OSDI questionnaire, and CFS. These subjects also obtained meibum samples, and then meibum chemokines (MIG, IFN-*γ*, IL-8, IP-10, and MCP-1) were examined and analyzed the correlations with ocular surface parameters.

**Results:**

MIG, IP-10, and MCP-1 were found clearly elevated in MGD subjects with higher MQ score than that in MGD subjects with low MQ score (MIG: *p* = 0.038, IP-10: *p* = 0.019, MCP-1: *p* = 0.040). The meibomian function was found mostly positively correlated with level of MIG (MGE: *r* = 0.600, *p* < 0.001; MQ: *r* = 0.579, *p* < 0.001) and IP-10 (MGE: *r* = 0.719, *p* < 0.001; MQ: *r* = 0.601, *p* < 0.001). The tear stability was found negatively correlated with the level of MIG (NIKBUT: *r* = −0.438, *p* = 0.005; LLT: *r* = −0.464, *p* = 0.003) and MCP-1 (NIKBUT: *r* = −0.425, *p* = 0.006; LLT: *r* = −0.761, *p* < 0.001). The OSDI was positively correlated with IL-8, IFN-*γ*, and MIG.

**Conclusion:**

Chemokines in meibum were significantly evaluated in MGD subjects suffering from severe meibomian gland quality. These findings indicate that chemokines play roles in the pathogenesis of MGD, and molecules targeted by chemokines may develop as novel agents for MGD therapy, perhaps through inhibiting inflammation in meibomian glands and microvascular in the eyelid margin.

## 1. Introduction

Meibomian gland dysfunction (MGD), a chronic abnormality of the meibomian glands, remarkably affects tear film stability and leads to various ocular surface disease problems [1]. The global prevalence of MGD was reported to range from 10 to 20%, while the prevalence of MGD in the Eastern Asian even achieved more than 50% [2]. In ophthalmology clinics, MGD is one of the most common disorders routinely, so it can be considered as a public health problem [3]. Normal meibomian glands can secrete clear oil meibum, which serve as a mixture of lipids via orifices as the outermost layer of the tear film [4]. The abnormal meibum which MGD patients produce becomes more stagnant than the normal meibum, which can lead to terminal meibomian gland duct obstruction and evaporative dry eye [1]. Although there are different pathogenic mechanisms responsible for DED owing to MGD, a common consensus is that the involvement of inflammation as an integral part of MGD and DED [5]. The malfunction of the meibomian gland leads to exacerbating of the meibum, and abnormal meibum further derangements of the ocular surface and triggers more inflammatory cytokine expression [6]. The vicious circle, named as “dry eye inflammatory vicious cycle”, forms between dry eye and inflammation [7], and abundant evidences identified that chronic inflammation on the ocular surface is located at the core pathogenesis of MGD [8].

In the DEWS II report, the etiology of MGD was described to be terminal duct obstruction impairing ocular surface homeostasis and leading to apparent inflammation and tear hyperosmolarity [9, 10]. Inflammation is an extremely complex process, and the chemotaxis process is a pivotal step in the promotion and regulation of the inflammatory response. Chemokine family, the most important factors among the chemotaxis process, is a group of low molecular weight proteins (between 8 and12 kDa) which can promote immune cell migration to act on the immune chemotaxis effect [11]. There are two large chemokine families, the C-X-C motif ligand (CXCL) and the C-C motif ligand (CCL) chemokine families, both of which can induce T cell infiltration and regulate autoimmune inflammation [12]. Once these chemokines are bound to the ligands, they can lead to tissue damage and clinical manifestations through polarizing the migration of specific immune cells and amplifying the inflammatory response.

Manuscripts focusing on tear inflammation in MGD are steadily increasing [13, 14], but there are rare reports concentrating on meibum chemokine examination in MGD patients. Considering that the core pathogenesis of MGD is located in the meibum secreted by the meibomian gland [1, 6], directly study on the inflammation cytokines in meibum has more explicit clinical value than inflammation cytokines in tears. The aim of the current study was to investigate the levels of different chemokines in meibum in MGD subjects. Furthermore, the correlations between meibum chemokines with ocular surface parameters were analyzed to seek possible meibum biomarkers for potential therapeutic agents in MGD.

## 2. Materials and Methods

### 2.1. Participants

This study was conducted in accordance with the tenets of the Declaration of Helsinki and was approved by the Institutional Review Board (IRB) of the Eye, Ear, Nose, and Throat (EENT) Hospital of Fudan University. The clinical trial was registered in the Chinese Clinical Trial Registry on June 2019. The registration number is ChiCTR-1900023732. Forty patients who experienced MGD were recruited from the EENT hospital from September 2019 to February 2020. According to the consensus of The International Workshop on Meibomian Gland Dysfunction, MGD was confirmed by meibomian gland function examination by the same ophthalmology [1, 15].

The inclusion criteria for MGD based on DEWS II are as follows: OSDI score > 12.5 points, FBUT < 10 s, and the presence of lid margin abnormalities, orifice abnormalities, and meibum abnormalities [1, 15]. These MGD patients aged above 18 years old, who voluntarily participated in the experiment. Subjects with immune related dry eye such as Sjogren's syndrome were excluded. Furthermore, subjects with certain ocular diseases (acute ocular inflammation, obvious scar or keratinization in the palpebral margin) or receiving physiotherapy for blepharitis (intense pulsed light, baby shampoo, and demodex blepharitis treatment) in the last 3 months may confound the study results; thus, they were excluded from the study. Subjects were also excluded if they had a related ocular surgery, including cataract surgery, trichiasis surgery, lachrymal duct obstruction, or refractive surgery in the past 3 months. After the procedure and potential consequences of the study were explained elaborately, informed consent was obtained from all subjects before the experiment.

Firstly, OSDI questionnaire was collected to evaluate the symptoms of MGD subjects. Subsequently, ocular parameters including tear meniscus height (TMH), noninvasive tear break-up time (NIKBUT), lipid layer thickness (LLT), incomplete blink rate (%), fluorescein tear film break-up time (FBUT), corneal fluorescein staining (CFS), Schirmer I test (SIT), meibomian gland expressibility (MGE), meibomian gland quality (MQ), lid margin, and meibograde. Finally, the meibum sample was collected and stored for further examination. Based on MQ examination, subjects with mild to severe abnormal meibum quality (ranging from cloudy with granular particulates to toothpaste-like particulates) were included into the MQ > 8 group, while subjects with slight abnormal meibum quality (ranging from clear oil to cloudy oil) were included into MQ ≤ 8 group.

### 2.2. Ocular Surface Parameters and OSDI

#### 2.2.1. OSDI

The OSDI questionnaire, containing a 12-item questionnaire with a scale of 0-100, has been designed to rapidly evaluate different ocular discomfort symptoms (soreness, light sensitiveness, and blurred vision). The OSDI questionnaire provides a rapid assessment of vision-related dyspraxia (difficulty reading, driving, operating a computer, and watching TV). There is a positive correlation between OSDI scores and the severity of ocular discomfort, with higher scores representing greater ocular discomfort [16].

#### 2.2.2. TMH and NIKBUT

TMH and NIKBUT were measured by an OCULUS Keratograph 5M (Wetzlar, Germany) equipped with modified TF-scan software. The procedure was repeated three times following the instructions of OCULUS Keratograph 5M by the same ophthalmology in a dark room. TMH was manually gauged at the central point of the lower lid margin on the images. Then, all participants were required to naturally blink twice, and then keep their eyes open as much time as possible until the next blink, the duration is defined as NIKBUT [17].

#### 2.2.3. LLT and Incomplete Blink Rate

LLT and incomplete blink rate were detected noninvasively by the LipiView® instrument (TearScience, Morrisville, NC, United States). All participants were instructed to blink naturally to record a 15 s video of the tear film interference pattern and analyze the LLT incomplete blink rate (%). The procedure was repeated twice times for each eye.

#### 2.2.4. FBUT

A fluorescein strip (Jingming) moistened with preservative-free saline gently touched the central lower lid margin. After participants blinked several times to ensure adequate coating of the complete cornea, they were required to rapidly open the eyes, and this point was recorded as the starting point (time = 0 sec). FBUT was defined as the interval between the starting point and the first black spot appearing in the stained team film with a cobalt blue filter and slit lamp microscope. The test was repeated three times and the average FBUT was calculated [16].

#### 2.2.5. CFS

The steps of corneal staining were similar to those for the assessment of the FBUT. The whole cornea was divided into five zones (central, superior, temporal, nasal, and inferior). Corneal epithelial injury was graded on a scale from 0 to 3: 0 if no epithelial injury; 1, <30 if corneal punctate stain; 2, >30 if corneal punctate stain but not fusion; and 3, if fusion of corneal staining or ulcer. The total CFS score ranged from 0 to 15.

#### 2.2.6. SIT

A sterile dry strip (Jingming®) was inserted into the lateral canthus of the lower eyelid away from the cornea for 5 min. The wetted length of the strip absorbed with tears was recorded as sitting to assess tear secretion. Potential SIT range is from 0 to 30 mm.

#### 2.2.7. Meibomian Gland Function

Meibomian gland function was evaluated using MGE score, MQ score, and lid margin score. The assessments were produced under slit lamp to grade MGE score, MQ score, and lid margin score. Five glands of the middle third of the upper lid were digitally pressed by MG evaluator (MGE-1000; TearScience), and the MGE was graded as 0-3: grade 0 if all five glands are expressible; grade 1, if 3-4 glands are expressible; grade 2, if 1-2 glands are expressible; and grade 3, if no glands are expressible [18]. Based on the phases of meibum, MQ was graded as follows: grade 0 if clear, grade 1 if cloudy, grade 2 if cloudy with granular particulates, and grade 3 if thick, like toothpaste-like particulates. Each of the eight glands of the lower eyelid was graded on a scale from 0 to 3. The scores of the eight glands were summarized (range: 0–24) [19]. According to the anomalous of the lid margin, lid margin score was graded as 0-4: grade 0 if absent of abnormal, and if present for any of the following parameters is recorded as 1: plugged meibomian gland orifices, vascular congestion, irregularity of the lid margin, and partly expression of the mucocutaneous borderline [20]. Combined with the upper and lower eyelid margins, the total score ranges from 0 to 8.

#### 2.2.8. Meibograde

Meibographies of the upper and lower eyelids were captured by the OCULUS Keratograph 5M (Wetzlar, Germany), and the meibomian gland dropout rate was analyzed qualitatively by ImageJ software (National Institutes of Health, USA). Meibograde of each eyelid was scored based on the meibomian gland dropout rate: 0 if meibomian gland area of loss = 0%, 1 if dropout rate less than 1/3 of the meibomian gland, 2 if dropout rate ranges from 1/3 to 2/3 of the meibomian gland, and 3 if dropout rate was over 2/3 of the meibomian gland. Meibogrades of the upper and lower eyelid were summed to a grade as 0-6 for each eye [21].

### 2.3. Meibum Inflammation Cytokines

#### 2.3.1. Meibum Samples Collection

A disposable 2.2 *μ*L collector (Seinda, Guangdong, China) was applied to obtain meibum samples at the orifice of the central meibomian glands of the upper and lower eyelid margin with meibomian massage simultaneously. The meibum samples were transferred into little microtubes immediately and then stored at -80°C for further assays.

#### 2.3.2. Assays for Inflammation Cytokines

MILLIPLEX MAP High Sensitivity T Cell Magnetic Bead Panel (Merck EMD Millipore, Billerica, MA, United States) for monokine induced by IFN-*γ* (MIG), interferon-gamma (IFN-*γ*), interleukin (IL)-8, interferon-inducible protein-10 (IP-10), and monocyte chemotactic protein-1 (MCP-1) was used according to the manufacturer's instructions. Luminex liquid suspension chip detection was performed using Huaying Biotechnologies (Shanghai, China). Briefly, meibum samples were incubated in microbead-embedded 96-well plates overnight at 4°C and subsequently incubated with the detection antibody for 1 h at room temperature at the next day. Next, Streptavidin-Phycoerythrin was added into each well of the plate and incubated for 30 min at room temperature, and the values were detected by a Luminex 200 system (Luminex Corporation, Austin, TX, United States).

### 2.4. Statistical Analyses

Data were analyzed using SPSS v.17.0 software (SPSS Inc.). Categorical data between the two groups were evaluated for statistical significance using the chi-square test. Continuous variables were presented as the mean ± standard deviation. The normal distribution test was performed to check whether the numerical variables were normally distributed. To compare the ocular parameters and levels of chemokine concentration, an independent sample *t*-test was used. The correlations between the ocular parameters and levels of chemokine concentration were performed using the Pearson rank correlation test. The results are indicated as *p* values, where *p* < 0.05 was considered to indicate a statistically significant difference.

## 3. Results

### 3.1. Demographic Data and Clinical Characteristics

Twenty MGD subjects whose MQ scores were above 8 with a mean age of 35.30 ± 9.71 years (14 females, 6 males) were enrolled and compared with twenty MGD subjects whose MQ scores were less than 8 (mean age: 34.35 ± 5.76 years; 13 females, 7 males). No significant differences in terms of sex (c2 = 0.109, *p* = 0.744) and age (*p* = 0.709) were found between the two groups. The demographic data and ocular surface parameters in MGD subjects with MQ > 8 and MGD with MQ ≤ 8 were listed in Table 1.

The meibomian gland function was comprehensively evaluated by MGE, MQ, meibograde, and lid margin. By comparing the meibomian gland function-related markers, subjects with more MQ scores showed more severe clinical manifestations than subjects with low MQ score (MGE: ^∗∗∗^*p* < 0.001, MQ: ^∗∗∗^*p* < 0.001, meibograde: ^∗∗∗^*p* < 0.001, lid margin: ^∗∗∗^*p* < 0.001; Table 1). NIKBUT, FBUT, and LLT are the major indexes of tear film stability. The values of the NIKBUT, FBUT, and LLT in MGD subjects with high MQ scores were significantly lower than those in MGD subjects with low MQ score (NIKBUT: ^∗∗∗^*p* < 0.001, FBUT: ^∗∗∗^*p* < 0.001, LLT: ^∗∗^*p* = 0.006; Table 1). The OSDI score in MQ > 8 subjects was mildly higher than that in subjects MQ ≤ 8 (^∗^*p* = 0.032). Tear secretion was assessed by SIT and TMH, and we found TMH and SIT was slightly higher in MQ > 8 subjects than that in subjects MQ ≤ 8. TMH showed a significant difference between the two group subjects (^∗^*p* = 0.029), while no significant difference in SIT was found (^∗^*p* = 0.086). The CFS and incomplete blink rate showed no significant differences between MGD subjects with high MQ scores and with low MQ scores.

### 3.2. Meibum Inflammation Cytokines

Five inflammatory cytokines (MIG, IFN-*γ*, IL-8, IP-10, and MCP-1) were examined and analyzed in meibum samples in both group subjects. Based on inflammatory cytokines results, MIG, IP-10, and MCP-1 were found clearly elevated in MGD subjects with high MQ score than that in MGD subjects with low MQ score (MIG: ^∗^*p* = 0.038, IP-10: ^∗^*p* = 0.019, MCP-1: ^∗^*p* = 0.040; Table 2). No significant differences of IFN-*γ* and IL-8 levels were found in meibum inflammation cytokines between the two group subjects (IFN-*γ*: *p* = 0.095, IL-8: *p* = 0.173; Table 2).

### 3.3. The Correlation between Meibum Cytokines and Ocular Surface Parameters

**Figure 1:**
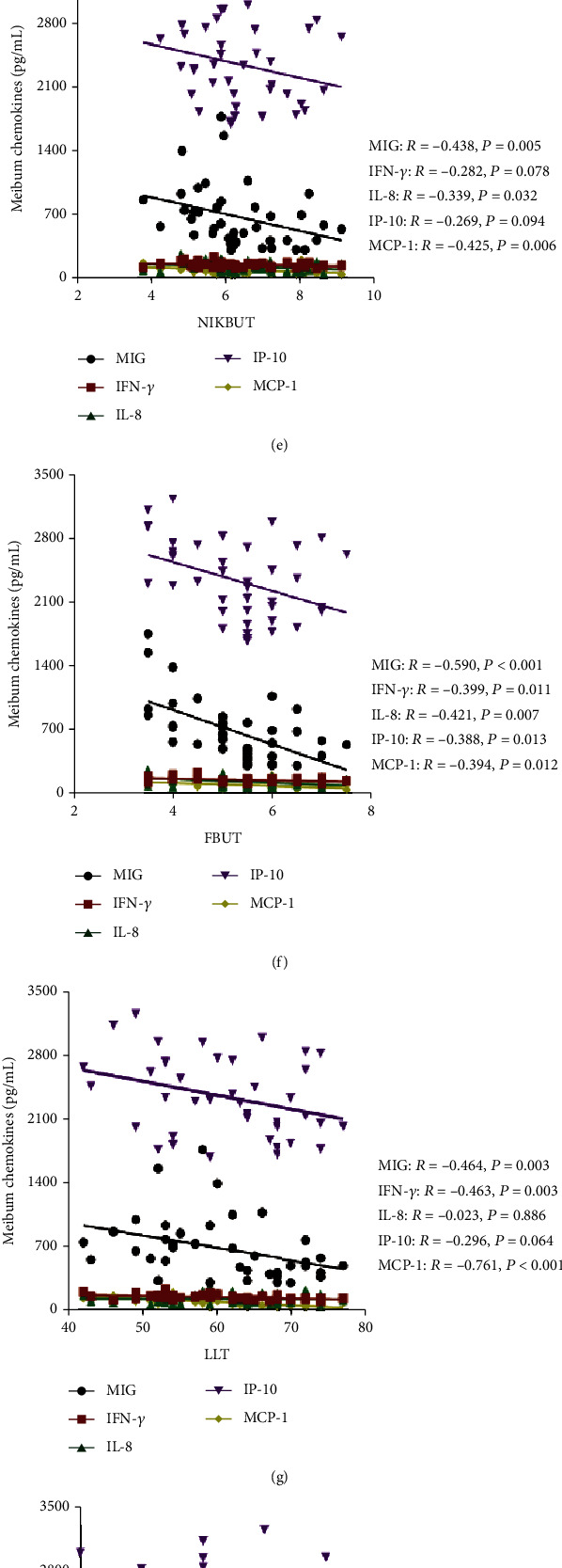
Correlation between ocular surface parameters and meibum inflammatory cytokines. (a) MGE score. The level of MIG (*r* = 0.600, ^∗^*p* = 0.015), IL-8 (*r* = 0.465, ^∗∗^*p* = 0.002), and IP-10 (*r* = 0.719, ^∗∗∗^*p* < 0.001) is positively correlated with the MGE score. No significant correlations were found between MGE and other inflammatory cytokines (all *p* > 0.05). (b) MQ score. The level of MIG (*r* = 0.579, ^∗∗∗^*p* < 0.001), IFN-*γ* (*r* = 0.321, ^∗^*p* = 0.044), IL-8 (r = 0.444, ^∗∗^*p* = 0.004), and IP-10 (*r* = 0.601, ^∗∗∗^*p* < 0.001) is positively correlated with the MQ score. No significant correlations were found between MQ and MCP-1 (*p* > 0.05). (c) Lid margin score. The level of MIG (*r* = 0.471, ^∗∗^*p* = 0.002), IP-10 (*r* = 0.569, ^∗∗∗^*p* < 0.001), and MCP-1 (*r* = 0.469, ^∗∗∗^*p* < 0.001) is positively correlated with the lid margin score. No significant correlations were found between lid margin and other inflammatory cytokines (all *p* > 0.05). (d) Meibograde score. The level of MIG (*r* = 0.382, ^∗^*p* = 0.015), IFN-*γ* (*r* = 0.489, ^∗∗^*p* = 0.001), and MCP-1 (*r* = 0.520, ^∗∗^*p* = 0.001) is positively correlated with the meibograde score. No significant correlations were found between meibograde score and other inflammatory cytokines (all *p* > 0.05). (e) NIKBUT. The level of MIG (*r* = −0.438, ^∗∗^*p* = 0.005), IL-8 (*r* = −0.339, ^∗^*p* = 0.032), and MCP-1 (*r* = −0.425, ^∗∗^*p* = 0.008) is negatively correlated with the NIKBUT. No significant correlations were found between NIKBUT and other inflammatory cytokine (all *p* > 0.05). (f) FBUT. The level of MIG (*r* = −0.590, ^∗∗∗^*p* < 0.001), IFN-*γ* (*r* = −0.399, ^∗^*p* = 0.011), IL-8 (r = -0.421, ^∗∗^*p* = 0.007), IP-10 (*r* = −0.388, ^∗^*p* = 0.013), and MCP-1 (*r* = −0.394, ^∗^*p* = 0.012) is negatively correlated with the FBUT. (g) LLT. The level of MIG (*r* = −0.464, ^∗∗^*p* = 0.003), IFN-*γ* (*r* = −0.463, ^∗^*p* = 0.044), and MCP-1 (*r* = −0.761, ^∗∗∗^*p* < 0.001) is negatively correlated with the LLT. No significant correlations were found between LLT and other inflammatory cytokines (all *p* > 0.05). (h) CFS score. No significant correlations were found between CFS scores and each inflammatory cytokine (all *p* > 0.05). (i) OSDI score. The level of MIG (*r* = 0.561, ^∗∗∗^*p* < 0.001), IFN-*γ* (*r* = 0.321, ^∗^*p* = 0.043), and IL-8 (*r* = 0.641, ^∗∗∗^*p* < 0.001) is positively correlated with the OSDI. No significant correlations were found between OSDI and other inflammatory cytokines (all *p* > 0.05). (j) TMH. The level of MIG (*r* = 0.324, ^∗^*p* = 0.041) is positively correlated with the TMH. No significant correlations were found between TMH and other inflammatory cytokines (all *p* > 0.05). (k) SIT. No significant correlations were found between SIT and each inflammatory cytokine (all *p* > 0.05). Significant differences between the correlations with meibum inflammatory cytokines and ocular surface parameters. The bold values mean significant results. ^∗^*p* < 0.05, ^∗∗^*p* < 0.01, ^∗∗∗^*p* < 0.001.

The meibum cytokines were identified to be significantly affecting meibomian gland function and tear film stability (Figure 1). The MGE was found positively correlated with the level of MIG (*r* = 0.600, ^∗∗∗^*p* < 0.001), IL-8 (*r* = 0.465, ^∗∗^*p* = 0.002), and IP-10 (*r* = 0.719, ^∗∗∗^*p* < 0.001) (Figure 1(a)). The MQ was found positively correlated with the level of MIG (*r* = 0.579, ^∗∗∗^*p* < 0.001), IFN-*γ* (*r* = 0.321, ^∗^*p* = 0.044), IL-8 (*r* = 0.444, ^∗∗^*p* = 0.004), and IP-10 (*r* = 0.601, ^∗∗∗^*p* < 0.001) (Figure 1(b)). The lid margin was found positively correlated with the level of MIG (*r* = 0.471, ^∗∗^*p* = 0.002), IP-10 (*r* = 0.569, ^∗∗∗^*p* < 0.001), and MCP-1 (*r* = 0.496, ^∗∗∗^*p* < 0.001) (Figure 1(c)). The meibograde was found positively correlated with the level of MIG (*r* = 0.471, ^∗∗^*p* = 0.002), IFN-*γ* (*r* = 0.489, ^∗∗^*p* = 0.001), IP-10 (*r* = 0.325, ^∗^*p* = 0.041), and MCP-1 (*r* = 0.520, ^∗∗^*p* = 0.001) (Figure 1(d)).

Moreover, meibum chemokines seriously affected the stability of the tear film. The NIKBUT was found negatively correlated with the level of MIG (*r* = −0.438, ^∗∗^*p* = 0.005), IL-8 (*r* = −0.339, ^∗^*p* = 0.032), and MCP-1 (*r* = −0.425, ^∗∗^*p* = 0.006) (Figure 1(e)). All five cytokines were found negatively correlated with FBUT (MIG: *r* = −0.590, ^∗∗∗^*p* < 0.001, IFN-*γ*: *r* = −0.399, ^∗^*p* = 0.011, IL-8: *r* = −0.421, ^∗∗^*p* = 0.007, IP-10: *r* = −0.388, ^∗^*p* = 0.013, and MCP-1: r = -0.394, ^∗^*p* = 0.012) (Figure 1(f)). The LLT was found negatively correlated with the level of MIG (*r* = −0.464, ^∗∗^*p* = 0.003), IFN-*γ* (*r* = −0.463, ^∗∗^*p* = 0.003), and MCP-1 (*r* = −0.716, ^∗∗∗^*p* <0.001) (Figure 1(g)). However, no significant correlation was found between CFS and five meibum cytokines.

In tear secretion and OSDI, the concentrations of meibum cytokines were also found partially correlated with these parameters. The OSDI was positively correlated with the level of MIG (*r* = 0.561, ^∗∗∗^*p* < 0.001), IFN-*γ* (*r* = 0.321, ^∗^*p* = 0.043), and IL-8 (*r* = 0.641, ^∗∗∗^*p* < 0.001) (Figure 1(i)). MIG was positively correlated with TMH (*r* = 0.324, ^∗^*p* = 0.041; Figure 1(j)), while no significant correlation was found between SIT and five meibum cytokines (Figure 1(k)).

## 4. Discussion

MGD is a chronic disease with a high level of prevalence among the human population and creates long-term damage to the ocular surface [3]. In consideration of the importance of inflammation in MGD, exploration and identification of the potential inflammatory cytokines that are specifically involved in the pathogenesis of MGD has a large significance. Once a particular cytokine in meibum was found to be tightly related to the severity of the disease, then the inhibitor or agonist targeted to the particular cytokine could be developed as a potential therapeutic agent of MGD. This is the original purpose of our research, and some meaningful data were obtained.

In the current study, the majority of ocular parameters in MQ > 8 group exhibited more serious clinical characteristics, and they were statistically significant in contrast to MQ < 8 group. About meibum inflammation cytokines, the levels of 3 meibum inflammation cytokines (MIG, IP-10, and MCP-1) of the 5 inflammatory cytokines we examined were largely elevated in MGD patients with worse meibum quality. Integrated with these two parts these results, a conclusion can be drawn that the increase of meibum inflammatory cytokines accompanied with more serious disease degree. A previous study [22] showed that increased levels of various inflammatory cytokines in tears (IL-6, IL-8, TNF-*α*, and IFN-*γ*) were found in MGD patients than normal people, and these inflammatory cytokines were also associated with meibomian gland function and tear stability. Another report [23] investigated the tear inflammation cytokines between normal subjects and MGD patients, and it was observed that the amount of inflammation cytokines (such as TNF-*α*, IL-1*β*, IL-6, IL-8, IL-12p70, and IFN-*γ*) was significant elevated in the tear of MGD patients, so inflammation may serve as the core characteristic in MGD patients. These reports verified our conclusion that worse meibomian gland function was closely associated with a higher level of tear inflammation cytokines. Thus, an inseparable relationship between the abnormalities of the meibomian glands is in association with ocular surface inflammation [9].

The 3 cytokines found robustly elevated in meibum are all classified to the chemokine family. MIG and IP-10 were included in CXCL family, while MCP-1 was included in CCL family. MIG/CXCL9 and IP-10/CXCL-10, also named “interferon-inducible CXC chemokine receptor 3 ligands”, are ELR-negative CXC chemokines induced by IFN-*γ* or other stimuli during infection or inflammation in several immune cell [24]. Several studies [25, 26] have demonstrated that they can effectively activate and recruit T lymphocytes to the target organ in vivo to exert immune chemotaxis function. In the current study, the increases in MIG and IP-10 levels were found positively correlated with worse meibomian gland function, while they were negatively correlated with tear stability. Combined the robust immunomodulatory effect of CXCL family (MIG/CXCL-9 and IP-10/CXCL-10) [27–29] with the highly expression of these two chemokines in MGD patients, we speculated that MIG and IP-10 play a critical role in the pathogenesis of MGD.

According to relevant studies [30], CXC chemokines were pointed out to be associated with neovascularization, and CXC chemokines which lack of ELR (MIG/CXCL-9, IP10/CXCL-10) are potent inhibitors of angiogenesis and microvascular. The antiangiogenesis effect of MIG and IP10 have been applied and certified in the treatment of various diseases [31, 32]. Neovascularization and microvascular in lid margin can be found among many MGD patients [6, 33], so the treatment targeted on neovascularization and microvascular (IPL) in lid margin has been proved to be effective in MGD patients [34]. Hence, we speculated that the increase of MIG and IP-10 in meibum treatment may potentially achieve the therapeutic effect on MGD by downregulating angiogenesis cytokines to inhibit neovascularization and microvascular in lid margin. Based on the transitional inflammatory and obvious neovascularization responses in MGD, systemic and topical treatment which can combine anti-inflammation with antiangiogenesis should be accepted in regular strategies to maintain the normal function of meibomian glands, and especially novel molecular targeted on MIG and/or IP-10 may have great prospects in clinical.

MCP-1, also named as CCL-2, is an inflammatory cytokine that is specific for monocyte chemotactic proteins and can bind to NF-*κ*B nuclear factor DNA to induce different immune cell migration to the designated tissue and trigger downstream signals to exert immune regulation functions [35, 36]. Our study has identified that MCP-1 was closely related with NIKBUT and FBUT, while not correlated with MGE and MQ. Compared to CCL chemokine family, we inferred that CXCL chemokines should be focused as the core pathogenesis of MGD. Based on the transitional expression of CXCL chemokines in meibum in MGD subjects, topical anti-CXCL-inflammation treatment have more potential to develop as novel strategies in the treatment of MGD.

Furthermore, there was no significant difference in the levels of IFN-*γ* and IL-8 between the two groups, but relationships were found between partially ocular parameters with these two cytokines in meibum. IL-8, also named as CXCL-8, is the most well-known molecule in the CXCL chemokine family, which has great attractive chemotactic effects on neutrophils, lymphocytes and basophils [37]. In contrast to normal people, IL-8 was found significantly elevated in tears in all kinds of dry eye patients, not just limited to MGD. Therefore, IL-8 may be positioned as the critical pathogenesis of various types of dry eye, and it strongly affects ocular discomfort symptoms [23, 38]. IL-8, as a vital member of the CXCL family, was confirmed to be related with MGE and MQ, thus the key role of CXCL family in maintaining normal meibomian gland function was confirmed once more. IFN-*γ*, the only member of type II interferon, is mainly secreted by natural killer cells (NK) and natural killer T cells (NKT) cells in the process of innate immunity and is also secreted by CD4 Th1 and CD8 cytotoxic T cells in the process of antigen-specific immunity [39]. Similar to IL-8, IFN-*γ* has also been proved to be significant elevated in most kinds of dry eye [23, 40]. OSDI, the only index to assess the subjective symptoms of dry eye, was found closely related to the levels of IL-8 and IFN-*γ*. Therefore, a combination therapy including anti-CXCL with anti-IL-8/anti-IFN-*γ* agent can simultaneously alleviate the ocular parameters and mitigate the discomfortable symptoms in MGD, to yield twice the result with half the effort.

Since the tear microenvironment is affected by various aspects, including tear quantity secreted by the lacrimal gland, meibum produced by the meibomian gland, and mucins secreted by the corneal and conjunctival epithelium [9], thus, the examination of the tear samples cannot be directly extrapolated to evaluate the function of meibomian gland. Meibum, as a noninvasive and convenient sample to obtain from meibomian glands directly, is the best carrier to reveal the microenvironment of meibomian glands. Therefore, the data obtained from meibum may be more accurate than the tear samples in the study of MGD. It is worth noting that meibum samples from MGD subjects were collected to more directly investigate the inflammation of meibomian glands, and we found that the expression of meibum inflammatory cytokines, especially chemokines (MIG, IP-10, and MCP-1) increased with the aggravation of MGD disease and can be detected as sensitive biomarkers in pre-evaluation the extent of MGD.

Due to the limitation of the small number of subjects, the inadequate number of inflammatory cytokines was also a deficiency in the current study. Furthermore, inflammatory cytokines have only been detected by Luminex chip without rechecked by ELISA assay, which is a shortcoming of the current study. Thus, further studies should be conducted with a larger number of participants examined with more analysis of meibum inflammatory cytokines using both Luminex chip and ELISA assay, to explicit illustrate more novel crucial cytokine biomarkers of MGD patients with strong evidence.

To the best of our knowledge, this study is the first to identify the chemokines in meibum samples, and we found that they were closely correlated with meibomian function and tear film instability. As the most direct and available carrier of meibomian gland secretion, the meibum sample is clearly more ideal for exploring the microenvironment of the meibomian glands than the tear sample. The current study provides a solid foundation for the technical support of meibum inflammatory cytokines in further meibomian gland inflammation research. Moreover, to prove that chemokines, especially CXCL family, play an important role in the pathogenesis of MGD, it can be well applied in developing novel therapeutic agents for MGD in clinical.

## Figures and Tables

**Table 1:** Demographic data and clinical characteristics of MQ > 8 and MQ ≤ 8. (Mean ± SD).

	MQ > 8 (*n* = 20)	MQ ≤ 8 (*n* = 20)	*p* value
Age (years)	35.30 ± 9.71	34.35 ± 5.76	0.709
F/M	14/6	13/7	0.744
MGE score	1.50 ± 0.51	0.75 ± 0.72	**<0.001**
MQ score	16.80 ± 3.14	5.80 ± 2.59	**<0.001**
Meibograde score	4.00 ± 1.45	1.75 ± 0.55	**<0.001**
Lid margin score	1.90 ± 0.64	0.65 ± 0.67	**<0.001**
NIKBUT (s)	5.657 ± 0.96	6.982 ± 1.20	**<0.001**
FBUT(s)	4.725 ± 0.98	5.875 ± 0.83	**<0.001**
LLT (nm)	57.05 ± 9.29	64.90 ± 7.77	**0.006**
CFS score	1.10 ± 1.17	0.90 ± 0.97	0.558
OSDI score	45.09 ± 15.92	34.63 ± 13.70	**0.032**
SIT (mm/5 minutes)	12.50 ± 3.98	10.15 ± 4.44	0.086
TMH (mm)	0.211 ± 0.05	0.181 ± 0.03	**0.029**
Incomplete blink rate (%)	51.50 ± 22.31	44.50 ± 22.12	0.325

MGE: meibomian gland expressibility; MQ: meibomian gland quality; NIKBUT: noninvasive keratograph, tear film break-up time; FBUT: fluorescein break-up time; LLT: lipid layer thickness; OSDI: Ocular Surface Disease Index; SIT: Schirmer TMH, tear meniscus height; CFS: fluorescein staining score. Significant differences between MQ > 8 and MQ ≤ 8 values. The bold values mean significant results. ^∗^*p* < 0.05, ^∗∗^ *p* < 0.01, ^∗∗∗^ *p* < 0.001.

**Table 2:** Comparison of meibum inflammation cytokines between MQ > 8 and MQ ≤ 8 (Mean ± SD).

	MQ > 8 (*n* = 20)	MQ ≤ 8 (*n* = 20)	*p* value
MIG (pg/mL)	768.40 ± 376.92	550.35 ± 251.40	**0.038**
IFN-*γ* (pg/mL)	150.46 ± 31.43	136.33 ± 19.43	0.095
IL-8 (pg/mL)	135.48 ± 50.17	112.97 ± 52.30	0.173
IP-10 (pg/mL)	2472.16 ± 356.81	2159.75 ± 446.96	**0.019**
MCP-1 (pg/mL)	101.82 ± 41.33	73.27 ± 43.74	**0.040**

MIG: monokine induced by IFN-*γ*; IFN-*γ*: interferon-gamma, IL-8: interleukin (IL)-8; IP-10: interferon-inducible protein-10; and MCP-1: monocyte chemotactic protein-1. Significant differences in inflammation cytokines between MQ > 8 and MQ ≤ 8 values. The bold values mean significant results. ^∗^*p* < 0.05, ^∗∗^*p* < 0.01, ^∗∗∗^*p* < 0.001.

## Data Availability

The datasets used and/or analyzed during the present study are available from the corresponding author on a reasonable request.

## Abbreviations

CFS: Corneal fluorescein staining
IPL: Intense pulsed light
MCP-1: Monocyte chemotactic protein-1
MGD: Meibomian gland dysfunction
MGE: Meibomian gland expressibility
MIG: Monokine induced by IFN-*γ*
MQ: Meibomian gland quality
OSDI: Ocular surface disease index
TMH: Tear meniscus height
NIKBUT: Noninvasive tear break-up time
FBUT: Fluorescein tear film break-up time
FL: Fluorescein staining
IFN-*γ*: Interferon-gamma
IL-8: Interleukin-8
IP-10: Interferon-inducible protein-10
LLT: Lipid layer thickness
SIT: Schirmer I test
TMH: Tear meniscus height.

## References

[1] Nelson J. D., Shimazaki J., Benitez-del-Castillo J. M. (2011). The international workshop on meibomian gland dysfunction: report of the definition and classification subcommittee. *Investigative Ophthalmology & Visual Science*.

[2] Stapleton F., Alves M., Bunya V. Y. (2017). TFOS DEWS II epidemiology report. *The Ocular Surface*.

[3] Craig J. P., Nelson J. D., Azar D. T. (2017). TFOS DEWS II report executive summary. *The Ocular Surface*.

[4] Nicolaides N., Kaitaranta J. K., Rawdah T. N., Macy J. I., Boswell FM 3rd, Smith R. E. (1981). Meibomian gland studies: comparison of steer and human lipids. *Investigative Ophthalmology & Visual Science*.

[5] Wei Y., Asbell P. A. (2014). The core mechanism of dry eye disease is inflammation. *Eye & Contact Lens*.

[6] Geerling G., Tauber J., Baudouin C. (2011). The international workshop on meibomian gland dysfunction: report of the subcommittee on management and treatment of meibomian gland dysfunction. *Investigative Ophthalmology & Visual Science*.

[7] Pflugfelder S. C., de Paiva C. S. (2017). The pathophysiology of dry eye disease: what we know and future directions for research. *Ophthalmology*.

[8] Lee H., Chung B., Kim K. S., Seo K. Y., Choi B. J., Kim T. I. (2014). Effects of topical loteprednol etabonate on tear cytokines and clinical outcomes in moderate and severe meibomian gland dysfunction: randomized clinical trial. *American Journal of Ophthalmology*.

[9] Willcox M. D. P., Argueso P., Georgiev G. A. (2017). TFOS DEWS II tear film report. *The Ocular Surface*.

[10] Bron A. J., de Paiva C. S., Chauhan S. K. (2017). TFOS DEWS II pathophysiology report. *The Ocular Surface*.

[11] Muller M., Carter S., Hofer M. J., Campbell I. L. (2010). Review: The chemokine receptor CXCR3 and its ligands CXCL9, CXCL10 and CXCL11 in neuroimmunity - a tale of conflict and conundrum. *Neuropathology and Applied Neurobiology*.

[12] Griffith J. W., Sokol C. L., Luster A. D. (2014). Chemokines and chemokine receptors: positioning cells for host defense and immunity. *Annual Review of Immunology*.

[13] Wei Y., Gadaria-Rathod N., Epstein S., Asbell P. (2013). Tear cytokine profile as a noninvasive biomarker of inflammation for ocular surface diseases: standard operating procedures. *Investigative Ophthalmology & Visual Science*.

[14] Liu R., Rong B., Tu P. (2017). Analysis of cytokine levels in tears and clinical correlations after intense pulsed light treating meibomian gland dysfunction. *American Journal of Ophthalmology*.

[15] Tomlinson A., Bron A. J., Korb D. R. (2011). The international workshop on meibomian gland dysfunction: report of the diagnosis subcommittee. *Investigative Ophthalmology & Visual Science*.

[16] Liu W., Gong L. (2021). Anti-demodectic effects of okra eyelid patch in demodexblepharitis compared with tea tree oil. *Experimental and Therapeutic Medicine*.

[17] Zhao S., Song N., Gong L. (2021). Changes of dry eye related markers and tear inflammatory cytokines after upper blepharoplasty. *Frontiers in Medicine*.

[18] Lee S. Y., Lee K., Park C. K. (2019). Meibomian gland dropout rate as a method to assess meibomian gland morphologic changes during use of preservative-containing or preservative-free topical prostaglandin analogues. *PLoS One*.

[19] Seo K. Y., Kang S. M., Ha D. Y., Chin H. S., Jung J. W. (2018). Long-term effects of intense pulsed light treatment on the ocular surface in patients with rosacea-associated meibomian gland dysfunction. *Contact Lens & Anterior Eye*.

[20] Nichols K. K., Foulks G. N., Bron A. J. (2011). The international workshop on meibomian gland dysfunction: executive summary. *Investigative Ophthalmology & Visual Science*.

[21] Zhao S., Duan J., Zhang J., Gong L. (2022). Evaluation of Meibomian gland function after therapy of eyelid tumors at palpebral margin with super pulse CO2 laser. *Disease Markers*.

[22] Lam H., Bleiden L., de Paiva C. S., Farley W., Stern M. E., Pflugfelder S. C. (2009). Tear cytokine profiles in dysfunctional tear syndrome. *American Journal of Ophthalmology*.

[23] Zhao H., Li Q., Ye M., Yu J. (2018). Tear Luminex analysis in dry eye patients. *Medical Science Monitor*.

[24] Luster A. D. (1998). Chemokines — Chemotactic Cytokines That Mediate Inflammation. *The New England Journal of Medicine*.

[25] Groom J. R., Luster A. D. (2011). CXCR3 ligands: redundant, collaborative and antagonistic functions. *Immunology and Cell Biology*.

[26] Van Raemdonck K., Van den Steen P. E., Liekens S., Van Damme J., Struyf S. (2015). CXCR3 ligands in disease and therapy. *Cytokine & Growth Factor Reviews*.

[27] Kuan W. P., Tam L. S., Wong C. K. (2010). CXCL 9 and CXCL 10 as sensitive markers of disease activity in patients with rheumatoid arthritis. *The Journal of Rheumatology*.

[28] Rosenblum J. M., Shimoda N., Schenk A. D. (2010). CXC chemokine ligand (CXCL) 9 and CXCL10 are antagonistic costimulation molecules during the priming of alloreactive T cell effectors. *Journal of Immunology*.

[29] Antonelli A., Ferrari S. M., Giuggioli D., Ferrannini E., Ferri C., Fallahi P. (2014). Chemokine (C-X-C motif) ligand (CXCL)10 in autoimmune diseases. *Autoimmunity Reviews*.

[30] Strieter R. M., Burdick M. D., Gomperts B. N., Belperio J. A., Keane M. P. (2005). CXC chemokines in angiogenesis. *Cytokine & Growth Factor Reviews*.

[31] Shen Q., Fan X., Jiang M., Ye Z., Zhou Y., Tan W. S. (2019). Inhibiting expression of Cxcl9 promotes angiogenesis in MSCs-HUVECs co-culture. *Archives of Biochemistry and Biophysics*.

[32] Carew J. S., Espitia C. M., Zhao W., Mita M. M., Mita A. C., Nawrocki S. T. (2017). Oncolytic reovirus inhibits angiogenesis through induction of CXCL10/IP-10 and abrogation of HIF activity in soft tissue sarcomas. *Oncotarget*.

[33] Knop E., Knop N., Millar T., Obata H., Sullivan D. A. (2011). The international workshop on meibomian gland dysfunction: report of the subcommittee on anatomy, physiology, and pathophysiology of the meibomian gland. *Investigative Ophthalmology & Visual Science*.

[34] Cote S., Zhang A. C., Ahmadzai V. (2020). Intense pulsed light (IPL) therapy for the treatment of meibomian gland dysfunction. *Cochrane Database of Systematic Reviews*.

[35] Bai X., Qi Z., Song G. (2015). Effects of monocyte chemotactic Protein-1 and nuclear factor of kappa B pathway in rejection of cardiac allograft in rat. *Transplantation Proceedings*.

[36] Ghoniem G., Farhan B., Csuka D., Zaldivar F. (2020). Potential role of monocyte chemoattractant Protein-1 in monitoring disease progression and response to treatment in overactive bladder patients. *International Neurourology Journal*.

[37] Russo R. C., Garcia C. C., Teixeira M. M., Amaral F. A. (2014). The CXCL8/IL-8 chemokine family and its receptors in inflammatory diseases. *Expert Review of Clinical Immunology*.

[38] Zhang C., Ding H., He H. (2021). Comparison of early changes in ocular surface markers and tear inflammatory mediators after femtosecond lenticule extraction and FS-LASIK. *International Journal of Ophthalmology*.

[39] Lee H., Min K., Kim E. K., Kim T. I. (2012). Minocycline controls clinical outcomes and inflammatory cytokines in moderate and severe meibomian gland dysfunction. *American Journal of Ophthalmology*.

[40] Coursey T. G., Bohat R., Barbosa F. L., Pflugfelder S. C., de Paiva C. S. (2014). Desiccating stress-induced chemokine expression in the epithelium is dependent on upregulation of NKG2D/RAE-1 and release of IFN-*γ* in experimental dry eye. *Journal of Immunology*.

